# Fluoride and Aluminium in Tea (*Camellia sinensis* L.)—Tea Quality Indicators and Risk Factors for Consumers

**DOI:** 10.3390/molecules28176396

**Published:** 2023-09-01

**Authors:** Anja Pavlovič, Gašper Tavčar, Maja Ponikvar-Svet

**Affiliations:** 1 Department of Inorganic Chemistry and Technology, Jožef Stefan Institute, Jamova 39, SI-1000 Ljubljana, Slovenia; gasper.tavcar@ijs.si; 2 Jožef Stefan International Postgraduate School, Jamova 39, SI-1000 Ljubljana, Slovenia

**Keywords:** aluminium, fluoride, tea quality, price, health risk

## Abstract

In recent years, the quality and sourcing of tea have gained importance in Europe, but information remains scarce. The aim of this study was to determine the concentrations of fluoride (F^–^) and total aluminium (Al) species in infusions of commercially available teas in Slovenia, and thus in Europe, and to relate them to tea quality and their impact on consumer safety. F^–^ concentrations were determined using a fluoride-ion-selective electrode and Al concentrations using inductively coupled plasma optical emission spectroscopy. A comparison of the results obtained for four selected tea samples using the calibration curve and a standard addition technique showed good agreement, with no interferences caused by the sample matrix. The concentrations of 35 commercial teas ranged from 0.34 to 4.79 and 0.51 to 8.90 mg/L for F^–^ and Al, respectively. The average concentrations of the two elements followed the same descending order: black filter > green filter > black leaves ≈ green leaves. Single and multivariate statistical methods supported the categorisation of teas by packaging but not by type, with tea in filter bags being more expensive than loose tea. The linear relationship between F^–^ and Al concentrations in infusions (*C*(Al) = 1.2134 · *C*(F^–^)) allows for the determination of one element and estimation of the other, leading to a significant reduction in laboratory effort and cost. This research advances tea assessment by proposing Al concentration alongside F^–^ as a quality indicator and provides the basis for tea-monitoring protocols. Finally, the daily consumption of larger quantities of tea (≈1 L) with elevated F^–^ and Al concentrations could potentially pose a health risk.

## 1. Introduction

“True” tea (*Camellia Sinensis* L.) is the most widely consumed beverage in the world, next to water [1]. Tea drinking has a long tradition and has been considered a health-promoting habit since ancient times. A marked increase in tea consumption due to emotional and family-related settings was observed during COVID-19 pandemic [2]. Tea is produced by processing the harvested leaves through a combination of drying and fermentation processes that determine the type of tea, i.e., its chemical composition, aroma, taste and colour [3]. The largest tea producers in the world are China and India [4], while the United Kingdom, Germany and Poland are the main tea importers within the European Union and act as important trade hubs [5]. The European countries with the highest per capita consumption of tea are Turkey (3.17 kg), Ireland (2.04), and the United Kingdom (1.63 kg), where black and green tea have the largest market shares [1,6].

The tea plant has the unique ability to absorb fluorine in large quantities from the soil without showing signs of toxicity. It has been suggested that the toxicity of fluorine to the plant is attenuated by the simultaneous uptake of aluminium [7,8], but the exact mechanism of detoxification in tea plant is still poorly understood. The fluorine and aluminium content in the plant is influenced by various environmental factors such as soil conditions, climate, altitude and precipitation. In addition, anthropogenic factors such as air and soil pollution caused by industrial activities and urbanisation, horticultural practises (including the application of organic and inorganic fertilisers, the use of pesticides and soil conditioners) and the quality of water used for irrigation can affect the content of these elements. Nevertheless, the greatest variations in the total content of fluorine and aluminium, which mainly accumulate in tea leaves, are associated with the age of the leaves, genetic varieties of the plant, the plucking method (mechanical or manual), and the duration of the wilting period [9,10,11].

Both fluorine and aluminium leach out during infusion, with fluorine mainly occurring in the form of fluoride ions (F^–^) and aluminium in different species (abbreviated as Al) such as Al-citrate, Al-oxalate, Al-polyphenol and Al-fluoride complexes [12,13,14]. Comparing results from different studies is difficult because the leaching efficiency of fluoride and aluminium depends on the method of infusion preparation (e.g., the amount of tea in relation to water, infusion time and temperature). In general, the F^–^ concentration in infusions of tea available in Europe typically ranges between 0.31 and 8.9 mg/L [15,16,17,18,19]. The Al concentration is higher, and can reach up to 20 mg/L [13,20,21,22,23]. Comparable concentrations are also reported in sparse studies investigating both F^–^ and Al concentrations in infusions [24,25,26,27]. Thus, the consumption of tea infusions may significantly contribute to the daily intake of both elements. It has been known for almost a century that fluoride ions have a beneficial effect against dental caries. Therefore, the European Food Safety Authority (EFSA) and many health authorities worldwide have set the adequate daily intake (ADI) of fluoride from all sources at 0.05 mg/kg body weight (bw) for children and adults [28,29]. However, the margins between the beneficial and deleterious effects of fluoride are narrow. A threshold of 0.03 mg/kg bw has been suggested for the occurrence of dental fluorosis [29]. A higher fluoride intake for a prolonged period of time can also affect various tissues and organs of the human body before the most obvious signs of fluoride poisoning appear, i.e., the development of dental fluorosis in children and skeletal fluorosis in children and adults [29]. In contrast, aluminium ions do not play a physiological role in metabolic processes, but can be toxic in high amounts, which is why EFSA set its tolerable weekly intake (TWI) at 1 mg/kg bw [30]. Chronic routine exposure to aluminium has been linked to diseases such as dialysis dementia, iron-induced microcytic anaemia, osteomalacia, and possibly Alzheimer’s disease [31,32]. The consumption of tea with high F^–^ concentrations may thus pose a health risk [33]. On the other hand, there is no clear consensus on the risks of Al associated with tea consumption. The increased toxicity of fluoride in the form of Al-fluoride complexes, which can form in infusions and mimic the chemical structure of a phosphate and impair the activity of phosphoryl transfer enzymes, is also a concern [29]. Therefore, the control of F^–^ and Al in tea is crucial; however, to date, the European Community has not provided threshold values for their content in tea leaves or infusions—the legislation regulating these values relates to foodstuffs such as fresh herbs and leaf vegetables [34].

The aim of this study was to expand the current knowledge on human exposure to fluoride and aluminium through the consumption of some popular green and black teas that are commercially available in Slovenia, and thus also in Europe. The tested hypothesis was that infusions of filter teas contain significant concentrations of fluoride and aluminium that can have adverse effects on human health. The main objectives of the study were: (1) to study the possible matrix effect of tea on the determination of fluoride concentration in tea infusions with fluoride-ion-selective electrode and the aluminium concentration with inductively coupled plasma optical emission spectroscopy using both a calibration curve and a multiple standard addition technique; (2) to compare the fluoride and aluminium concentrations in infusions with respect to the manufactured form or type of tea; (3) to investigate the relationship between fluoride and aluminium concentrations; (4) to examine the price of tea in relation to the manufactured form and type; (5) to assess the cumulative exposure to fluoride and aluminium from tea and diet and the potential health risks.

## 2. Results and Discussion

### 2.1. Concentration of Fluoride and Aluminium in Tea Infusions

Fluoride and aluminium leach from the tea during infusion. A number of studies report concentrations of F^–^ and Al in tea infusions. However, the results are difficult to compare because the efficacy of the leaching greatly depends on the method of preparation of the infusion, e.g., the amount of tea in relation to water, infusion time and temperature, and differences in analytical procedures. The procedure used in this study was therefore adapted to mimic a domestic manner of preparing the tea infusion. The F^–^ concentration in our study was determined with a fluoride-ion-selective electrode (ISE) and the Al concentration was determined with inductively coupled plasma optical emission spectroscopy (ICP-OES). The calibration curve technique is the most widely used calibration method. However, the tea infusion matrix is very complex and therefore challenging, i.e., the matrix can significantly affect the stability and performance characteristics of inductively coupled plasma and the Al present in the sample matrix can interfere with the determination of F^–^ with ISE. The repeatability of the entire procedure for F^–^ and Al determination was therefore investigated on four randomly selected tea samples (one from each group) using the calibration curve and the standard addition technique. The latter is the technique of choice when it is not possible to match the samples to the standards. Samples (BF1, BL5, GF2, GL3) were weighed into commercial filter bags to ensure that all samples were prepared using the same procedure. The analysis also encompassed empty commercial filter bags, while the original tea bags in which the teas were initially packaged were excluded due to the potential presence of tea remnants containing F^–^ and Al. The corresponding results are presented in Table 1.

The results of the measurements of the two elements in infusions with the calibration curve and the standard addition technique were in good agreement. No significant difference was found between the average concentrations when a *t*-test was used to compare two experimental means. Good repeatability of the measurements with a relative standard deviation (RSD) of generally less than ± 9% for both elements was demonstrated without interferences caused by the sample matrix. The commercial filter bags used in the experiments were analysed according to the same procedure. The F^–^ and Al concentrations were below the limit of detection (LOD) (0.013 µg/mL for F^–^ and 0.0013 µg/mL for Al, presented in Appendix A).

Based on these experiments, the calibration curve technique was chosen for both elements, because of the slightly better repeatability of the F^–^ analysis and the shorter analysis time for both elements compared to the standard addition technique. The concentrations of F^–^ and Al determined in infusions of 35 common teas available in Slovenia, as well as their origin and prices, are listed in Table 2.

### 2.2. Fluoride and Aluminium Concentrations in Infusions with Respect to the Form and Type of Tea

**Figure 1:**
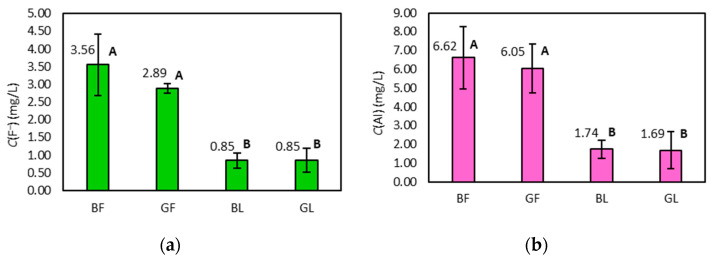
Average concentrations of fluoride (**a**) and aluminium (**b**) in infusions (± SD) of tea, categorised according to the type and packaging. Average values that do not have a common letter are significantly different (*p* < 0.05).

The results presented in Table 2 show that the concentration of F^–^ and Al in infusions ranges from 0.34–4.79 mg/L and 0.51–8.90 mg/L, respectively. The determined concentrations are in agreement with previously published results for tea available in Europe [13,15,16,17,18,19,20,21,22,23]. The average concentrations of both elements in infusions of tea, categorised by type and packaging, follow the same descending order: black filter > green filter > black leaves ≈ green leaves (Figure 1).

The average F^–^ and Al concentration in infusions of filter tea is significantly higher than that in infusions prepared from leaves according to the results of the Tukey test (Figure 1). The difference in the average concentration of green and black tea infusions is not significant.

**Figure 2:**
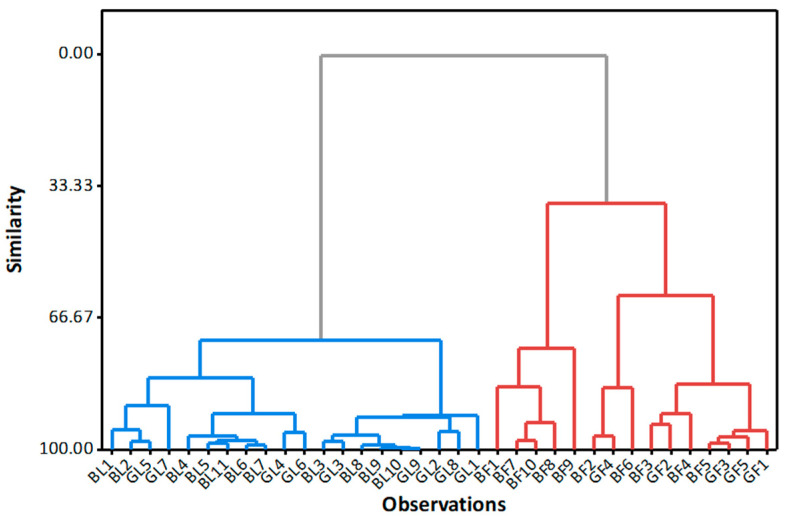
Dendrogram of cluster analysis with complete linkage and Euclidean distance method for 35 tea samples based on fluoride and aluminium concentrations in infusions.

The categorisation of teas by packaging but not by type is also supported by the results of the multivariate analysis using the hierarchical cluster method, where F^–^ and Al concentrations in infusions were used as independent variables (Figure 2).

The results of the cluster analysis show the formation of two clusters, i.e., a cluster for teas in filter bags and a separate cluster for teas in loose form. No separate clusters were formed for black and green tea.

The categorisation of teas in terms of packaging form based on F^–^ or Al concentration in the infusion is consistent with previously reported results for teas available in Europe [15,16,17,19,22]. One conceivable explanation for this difference is that the teas in filter bags are produced from inexpensive raw material—the paper bag masks a low-cost material, while flavourings and essences enhance their fragrance [15,35]. No significant difference between green and black teas based on F^–^ and Al concentration in infusions was observed. However, differentiation according to type is less reliable than differentiation according to form [15,16,18,19,21]. The increased Al content in black tea may be attributed to the frying of the leaves, which is thought to stop the fermentation process and is carried out with Al–Cu alloy pans [26]. In addition, the elemental composition of black tea was suggested to be higher than that of green tea leaves—black tea leaves are air-dried, while green leaves are steamed with water vapour, which can lead to losses [36]. However, a recent article suggests that geographical origin is the main reason for the clustering of tea samples and that different treatment processes (black or green tea) do not significantly affect the mineral composition of the leaves [37]. In this case, the aluminium-to-fluoride molar ratio in infusions of green and black tea should be comparable.

### 2.3. Relationship between Fluoride and Aluminium Concentrations in Tea Infusions

**Figure 3:**
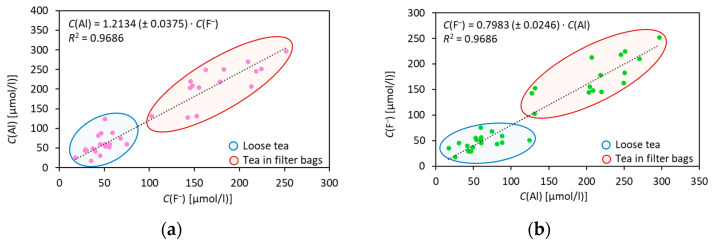
Concentrations of aluminium in tea infusions as a function of fluoride concentration (**a**) and fluoride in tea infusions as a function of aluminium concentration (**b**).

Fluoride and aluminium are stored in the vacuoles of tea leaves, where F^–^ is present in the form of complexes with aluminium, iron and calcium ions [14], while Al is also bound to catechins, phenols and organic acids [12,38]. Additionally, aluminium is also present in cell walls, where it is bound to pectin and hemicellulose [38]. Fluoride and aluminium leach out during infusion and their relationship can be described by linear functions forced through the origin (Equations (1) and (2), Figure 3). The decision to force the functions through the origin was made because the *p*-values of the constant terms of the linear equations (*C*(Al) = *f* (*C*(F^–^)), *p* = 0.379 and *C*(F^–^) = *f* (*C*(Al)), *p* = 0.554) were above the alpha level of 0.05, indicating that the terms were not statistically significant.

*C*(Al) = 1.2134 · *C*(F^–^), *R*^2^ = 0.9686 (1)

*C*(F^–^) = 0.7983 · *C*(Al), *R*^2^ = 0.9686 (2)

The data presented in Figure 3 support the categorisation of teas based on their Al and/or F^–^ concentrations in infusions with respect to their packaging into two groups. This is consistent with the results of the Tukey test and cluster analysis. The slopes of the fitted curves with a high squared linear regression coefficient (*R*^2^ > 0.96) represent the molar ratio of Al: F^–^ (Figure 3a) and of F^–^: Al (Figure 3b). The precision of the slope of both curves suggests that the leaching efficacy of fluoride and aluminium from the tea into the infusion is not affected by the localisation of fluoride and aluminiuim in different cell organelles, tea types or packaging. Thus, the fermentation process has little effect on the Al: F^–^ ratio, i.e., on the change in the mineral composition of the tea leaves. It is the geographical origin that most likely plays a key role in the mineral composition of tea [37].

The molar ratio between Al and F^–^ in infusions, which is greater than 1, is consistent with previous studies from Brazil [25] and China [26]. Moreover, an Al: F^–^ molar ratio greater than 1 is evident if comparing the results of the studies in which either F^–^ or Al concentrations in infusions were determined [13,15,16,17,18,19,20,21,22,23]. Our results, however, contradict those reported in Europe, where an Al: F^–^ molar ratio of less than 1, i.e., 0.59, was reported [24]. This low ratio could be a consequence of fluoride interference in the spectrophotometric determination of aluminium. In another study, a wide range of Al: F^–^ molar ratios between 0.04 and 6.04 was reported [27]. In this study, infusions of teas in filter bags were prepared differently from infusions of loose tea. In addition, the capacity of the total ionic strenght adjustement buffer (TISAB) used for Al complexation in the potentiometric determination of F^–^ by fluoride ISE was probably too weak [27,39].

Fluoride and aluminium leaching into the infusion are evidently linked, although the exact mechanism of their leaching has not yet been elucidated. The linear correlation between the concentrations of F^–^ and Al enables the determination of one element, facilitating the estimation of the other. This approach substantially minimizes the need for extensive laboratory work and costs. This is crucial because fluoride was previously proposed for the quality assessment of tea products from certain sources [40]. Our findings further establish that the aluminium concentration in tea infusions can serve as a reliable indicator of tea quality. Furthermore, the F^–^ and Al content in tea is also related to the quality of the tea in terms of total polyphenol and amino acid content [11,40,41]. Thus, the F^–^ (and/or Al) concentration in tea infusions can be considered a key parameter for tea quality and the methods used in this study could serve as a starting point for the development of appropriate protocols for a rapid and simple screening of tea quality.

### 2.4. Price Performance

**Figure 4:**
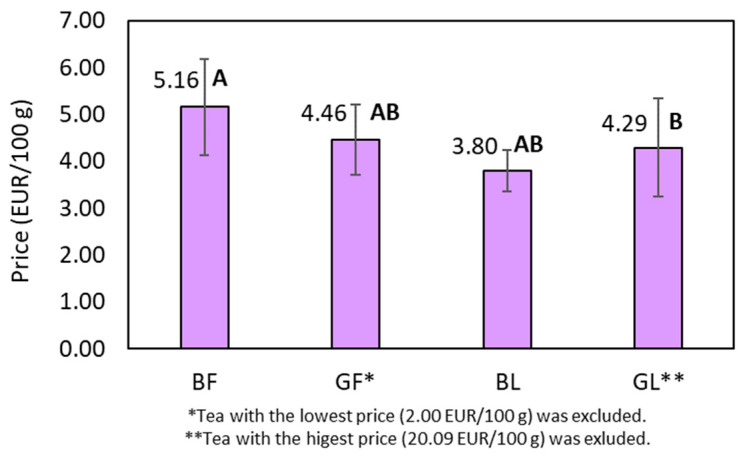
Categorisation of teas according to the form and type and average price. Averages that do not have a common letter are significantly different (*p* < 0.05).

The market share of tea packaged in filter bags is expected to show the highest growth rate compared to alternative packaging methods, including loose teas. This growth can be attributed to the ease of preparation and disposal associated with filter bags [42]. In the present study, wide price ranges were found, ranging from 2.00 EUR/100 g for commercial green tea in filter bags to 20.09 EUR/100 g for premium loose green tea (Table 2). The average prices of the teas, categorised by form and type, were compared using the Games–Howell test for normally distributed data with unequal variances (Figure 4).

The results presented in Figure 4 show a statistically significant price difference between black tea in filter bags and black tea in loose form, with loose tea being cheaper on average. Furthermore, determining the geographical origin of teas in filter bags proves to be a challenge. In contrast to loose-tea counterparts, there was no origin-specific information on the filter tea samples examined in this study (see Table 2). These findings seem to contradict the generally accepted notion that higher-quality products are usually more expensive and their origin is easier to trace. A reasonable explanation for the observed discrepancy is the packaging and processing costs associated with tea in filter bags, which require additional materials and machine-processing to make attractive and visually appealing packaging. Additionally, a tea pack typically contains 30–40 g of tea (20 filter bags of 1.5–2 g of tea each), while loose-leaf teas are usually packed in larger quantities, e.g., 100 or 500 g per container. This adds to the perception that tea in filter bags is cheaper than loose tea. From the available information, we can conclude that the price of tea and its quality are not necessarily correlated. This is in accordance with a recently reported model on consumer attitudes towards high-price organic tea [43]. Furthermore, other factors, such as the origin, growing and processing, transportation and global demand for tea can also influence the pricing of tea products.

Due to the lower fluoride and aluminium concentration, comparable or even lower price, and reduction in packaging material and waste, one can conclude that tea in loose form should be the first choice for a consumer. Nevertheless, the quality of tea in general, and tea in filter bags in particular, should be carefully monitored to ensure consumer safety and environmental well-being.

### 2.5. Exposure to Fluoride and Aluminium through Tea

The adequate daily intake (ADI) for fluoride from all sources is set by EFSA and many health authorities worldwide at 0.05 mg/kg bw [28,29]. However, the evidence suggests that the margin between beneficial and harmful effects is narrow [29]. The available data for aluminium do not allow for any dose–response relationship to be established. Due to the cumulative nature of aluminium in the body after dietary intake, the tolerable weekly intake with diet, including water and beverages (TWI), was set at 1 mg/kg body weight [30]. The possible formation of Al–fluoride complexes, which resemble a phosphate group and are more toxic than fluoride or aluminium alone, is also a concern [29].

Diet is an important source of fluoride and aluminium, and tea can contribute significantly to the intake of both [29,30]. The bioavailability of fluoride from tea infusions is close to 100% [33]. The low bioavailability of Al (0.1–0.3% [12,30]) is increased in the presence of fluoride [30,44]. Knowing the risk of exposure to F^–^ and Al or their combination is therefore crucial to avoid possible health effects.

**Figure 5:**
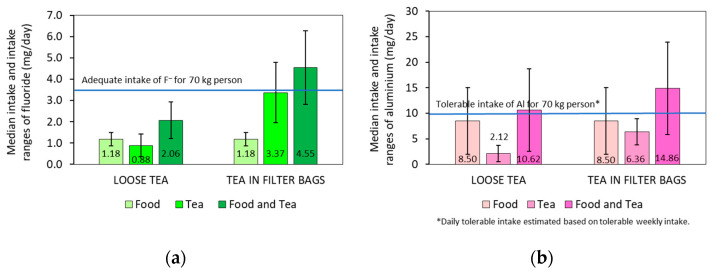
Estimated medians and ranges of daily fluoride (**a**) and aluminium (**b**) intakes from diet and tea for a 70 kg person in non-fluoridated areas of Europe.

Direct methods of monitoring nutrient intake are preferable to indirect methods using biomarkers, which are often influenced by factors such as absorption and metabolism [45]. In this study, daily intakes of fluoride and aluminium from food and tea in Europe were estimated based on data obtained using the direct methods from the current and previous studies [29,30]. In the absence of specific data on average daily aluminium intake, median daily intakes from food and tea were calculated based on intake ranges for both elements for a standardised weight of 70 kg (Figure 5).

Tea significantly contributes to the daily intake of fluoride and aluminium. For adults weighing 70 kg, the consumption of 1 L of tea can account for 25% of the ADI for fluoride for loose tea and 96% for filter tea infusion (Figure 5a). In the case of aluminium, TWI was first converted into a tolerable daily intake (TDI). For adults weighing 70 kg, the consumption of 1 L of tea can account for 21% of the TDI for aluminium for loose tea and 64% for filter tea infusion (Figure 5b). Thus, the consumption of 1 L of tea in filter bags on a normal diet can easily lead to an adult exceeding the ADI for F^–^ and the TDI for Al. In fluoridated areas (e.g., some parts of Ireland, the United Kingdom, the United States and Canada) or in areas with a high natural fluoride concentration in drinking water, these intake levels are even higher.

In Europe, the range of per-capita consumption of tea in 2020 was very wide. It was highest in Turkey (3.07 kg), followed by Ireland (2.04 kg) and the United Kingdom (1.63 kg). It was lowest in Serbia (0.01 kg) [1]. However, per capita consumption is a statistical figure that does not take into account that tea is often consumed in a chronic fashion. Therefore, excessive tea consumption could pose a potential risk for health problems related to fluoride and aluminium exposure among Europeans who consume larger amounts of tea. However, to date, the European Community has not set limits on the fluoride or aluminium content of tea leaves or tea infusions—the legislation that regulates these values refers to foodstuffs such as fresh herbs and leafy vegetables.

## 3. Materials and Methods

### 3.1. Reagents and Materials

All reagents were of analytical quality. Ultrapure (Milli-Q) water (18.2 MΩ cm) from a Direct-Q 3 UV system (Merck Millipore, Darmstadt, Germany) was used for all steps of sample preparation and analysis. Conc. HNO_3_ was purchased from Merck (Darmstadt, Germany). A citrate buffer solution (CBS) was prepared as previously described [46].

Working standards for fluoride (F^–^) were prepared from a (100.0 ± 0.5) mg/L-certified standard solution (Thermo Fisher Scientific, Waltham, MA, USA). A Merck-IV-certified ICP multi-element standard solution ((1001 ± 10) mg/L, aluminium) was used to prepare working standards for aluminium (Al). A 0.050 mol/L working solution of sodium fluoride was prepared by dissolving NaF (Merck) in water.

The accuracy of the calibration curve for F^–^ was checked with another certified F^–^ standard solution and for Al with an Inorganic Ventures certified-quality standard IV-ICPMS-71A ((10.01 ± 0.04) mg/L, aluminium) (Christiansburg, VA, USA).

Samples were weighed into brown tea filter Cilia size M (Melitta, Minden, Germany). The 0.45 µm polytetrafluoroethylene hydrophobic membrane filters (25 mm diameter) from Machery Nagel (Dueren, Germany) were used for filtration with a 10 mL syringe Chirana (Stará Turá, Slovakia).

### 3.2. Instrumentation

A Mettler XSR225DU analytical balance (Mettler Toledo, Zürich, Switzerland) was used for weighing. F^–^ concentrations were potentiometrically determined on a Metrohm 906 Titrando system (Herisau, Switzerland) equipped with a temperature sensor (Metrohm) with a Thermo Fisher Scientific Orion 96-09 combined ion-selective electrode (ISE). Al concentrations were determined by inductively coupled plasma optical emission spectroscopy (ICP-OES) on an Agilent (Agilent Technologies Inc., Tokyo, Japan) 5800 VDV instrument equipped with a concentric OneNeb nebulizer mounted onto a single-pass glass cyclonic spray chamber.

### 3.3. Sample Collection and Preparation

Fluoride and aluminium concentrations were determined in infusions of common green (14) and black (21) teas available in Slovenia and most European countries. The teas in filter bags were purchased in commercial shops and the loose teas in teahouses in 2022. The teas were divided into four main groups according to packaging and type: black filter teas (BF), black loose teas (BL), green filter teas (GF) and green loose teas (GL). The characteristics of the teas studied are listed in Table 2.

Samples were prepared for analysis according to the previously reported procedure [16]. A total of 1 g of the homogenised tea was weighed into a filter bag to the nearest 0.1 mg. The bag was infused with 100 mL of boiling water, covered with a lid and left to stand (*t* = 5 min) before the filter bag was removed.

### 3.4. Determination of Fluoride

The F^–^ concentration in the infusions was determined with fluoride ISE according to the previously described method, with slight modifications [47,48]. In brief, the F^–^ concentration was determined at room temperature in a 5 mL sample aliquot, which was spiked by adding F^–^ concentration of 0.20 µg/mL to bring the concentration in low-level samples within the linear working range of the ISE. To ensure constant ionic strength, adequate pH and complexation and masking of interfering ions (mainly aluminium [39]), 25 mL of CBS buffer was added to the samples and standards and diluted to 50 mL with water. Fluoride in infusions of empty filter bags was determined in 25 mL sample aliquot.

#### 3.4.1. Calibration Curve Technique

The electrode was calibrated at 0.20 and 1.0 μg/mL F^–^. The accuracy of the calibration curve was checked with a 0.20 μg/mL F^–^ solution prepared from a standard solution that differed from the one used for calibration [48]. The deviation of the potential measured in this solution was not allowed to exceed ± 0.2 mV, which corresponds to approximately ± 0.04 μg F^–^ per 50 mL solution. Three successive measurements were made, the average of which was subtracted from the measurement results of the samples. The accuracy of the calibration curve was checked every two hours with another certified standard solution.

#### 3.4.2. Standard Addition Technique

F^–^ concentration was determined by a multiple standard addition technique using a 0.050 mol/l working solution of NaF [48]. At least six consecutive measurements of 0.20 μg/mL F^–^ standard solution were performed. Their average value was subtracted from the results of the samples. The F^–^ concentration in the blank sample was checked every two hours and the result was required to be within the range of the average ± SD.

### 3.5. Determination of Aluminium+

The Al concentration in the infusions was determined with ICP–OES in axial view. The instrument was operated under the following conditions: RF power 1.2 kW; plasma gas 12.0 L/min; auxiliary gas 1.0 L/min; nebulizing gas of 0.70 L/min; sample flow rate 0.75 mL/min; stabilisation and sample uptake delays 15 and 25 s; rinse and replicate times 25 and 5 s, respectively; and number of replicates 6. The mean background-corrected intensity of the Al 396.152 nm line [13] was used for calibration. The aluminium concentration was determined in an aliquot of 1 mL of the sample, which was acidified with 1.2 mL conc. HNO_3_ before dilution to 50 mL. Infusions of filter bags were analysed without dilution directly after acidification.

#### 3.5.1. Calibration Curve Technique

The working standard solutions between 0.0002 and 5 μg/mL used for the 13-point calibration were prepared by successive dilution of a certified standard solution. Another certified reference material was used to check the accuracy of the calibration curve.

#### 3.5.2. Standard Addition Technique

Aliquots of the sample solution were spiked with a multi-element standard and acidified with conc. HNO_3_ before being diluted to 50 mL. The added concentrations of aluminium were 0, 0.05, 0.1, 0.5 and 1 μg/mL.

### 3.6. Statistical Analysis

Minitab 17.1.0.0. software (Champaign, IL, USA) was used for statistical analysis. The analysis was performed on at least duplicate sample portions. The concentrations of F^–^ and Al in each portion were measured at least twice. Data are presented as average ± SD, and significance was accepted at *p* < 0.05. Averages were compared using a *t*-test for comparison of two experimental means. Cluster analysis was performed using the Euclidean distance method. The significance of differences between groups was tested using analysis of variance (ANOVA) followed by Tukey’s test. In cases with unequal variance, the non-parametric Games–Howell test was performed.

## 4. Conclusions

The average F^–^ and Al concentration in infusions of tea in filter bags is significantly higher than in infusions from leaves, but the difference in the average concentration of infusions of green and black tea is not significant. The F^–^ concentration in relation to the Al concentration in the infusion can be described by a linear function and both can be used as indicators of tea quality. According to our results, the price of loose tea is significantly lower than that of filter tea. Hence, loose tea should be the first choice for a consumer due to its lower fluoride and aluminium concentration, lower price and reduced packaging material. Tea is often consumed chronically and a larger consumption (1 L) of tea with F^–^ and Al concentrations, as found in this study, could pose a potential health risk.

In conclusion, the monitoring of tea quality in Europe should be made mandatory by European authorities. Food and beverage manufacturers, including tea manufacturers, should be required by regulation to comply with labelling guidelines that clearly indicate the presence and quantity of F^–^ and Al in their products. To streamline the process and minimise costs, standardised analytical methods should be introduced, such as the methods proposed in this study.

## Figures and Tables

**Table 1:** The determined fluoride and aluminium concentrations in tea infusions in four selected tea samples using calibration curve and standard addition technique.

Sample	*n* ^1^	*C*(F^–^) (mg/L) ^2^		*C*(Al) (mg/L) ^2^	
		Calibration Curve	Standard Addition	Calibration Curve	Standard Addition
BF1	3	4.05 ± 0.03	4.04 ± 0.22	6.21 ± 0.20	6.12 ± 0.08
BL5	3	0.96 ± 0.03	0.96 ± 0.08	1.79 ± 0.05	1.81 ± 0.06
GF2	3	3.08 ± 0.02	3.16 ± 0.18	7.48 ± 0.12	7.52 ± 0.17
GL3	3	0.71 ± 0.02	0.72 ± 0.03	1.48 ± 0.07	1.52 ± 0.07

^1^ Number of parallel determinations. ^2^ Values are averages ± standard deviation (SD).

**Table 2:** The determined fluoride and aluminium concentrations in tea infusions using calibration curve technique and the price of the tea.

Symbol, Trade Name, Origin ^1^	*n* ^2^	*C*(F^–^) (mg/L) ^3^	*C*(Al) (mg/L) ^3^	Price (EUR/100 g)
Black filter (BF) tea				
BF1, English Breakfast, N/A ^4^	3	4.05 ± 0.03	6.21 ± 0.20	6.10
BF2, English Breakfast, N/A	2	2.71 ± 0.04	3.83 ± 0.02	5.00
BF3, English Breakfast Supreme, N/A	2	3.47 ± 0.15	7.51 ± 0.25	5.28
BF4, Gold, N/A	2	3.39 ± 0.05	6.56 ± 0.23	3.38
BF5, Prince of Wales, N/A	2	2.81 ± 0.19	6.25 ± 0.28	6.74
BF6, English Tea No.1, N/A	2	1.95 ± 0.03	3.93 ± 0.06	5.00
BF7, Earl Grey, N/A	2	4.15 ± 0.07	7.38 ± 0.18	6.38
BF8, Earl Grey, N/A	2	3.99 ± 0.17	8.11 ± 0.28	4.09
BF9, Earl Grey Royal, N/A	2	4.79 ± 0.07	8.90 ± 0.05	4.91
BF10, Irish Cream, N/A	2	4.26 ± 0.03	7.53 ± 0.30	4.76
Black loose (BL) tea				
BL1, English Breakfast, India	2	1.12 ± 0.07	2.65 ± 0.11	3.29
BL2, Irish Morning, Sri Lanka	2	0.88 ± 0.01	2.63 ± 0.16	3.65
BL3, Earl Grey Decaffeine, Sri Lanka	2	0.76 ± 0.03	1.25 ± 0.01	4.20
BL4, Earl Grey Superior, India	2	0.86 ± 0.04	1.80 ± 0.02	4.20
BL5, Earl Grey, Sri Lanka	3	0.96 ± 0.03	1.79 ± 0.05	3.65
BL6, Keemun, China	2	1.05 ± 0.07	1.59 ± 0.03	3.11
BL7, Yunnan, China	2	0.97 ± 0.08	1.63 ± 0.16	3.65
BL8, Ceylon OP1 Kenilworth, Sri Lanka	2	0.55 ± 0.01	1.41 ± 0.02	3.65
BL9, Assam TGFOP1 Dirial, India	2	0.54 ± 0.01	1.31 ± 0.01	3.65
BL10, Darjeeling TGFOP1, India	2	0.58 ± 0.01	1.29 ± 0.02	4.57
BL11, Bio Black Tea Darjeeling Premium, India	2	1.06 ± 0.02	1.80 ± 0.09	4.21
Green filter (GF) tea				
GF1, Green Tea, N/A	2	2.77 ± 0.07	6.58 ± 0.18	3.86
GF2, Green Tea, N/A	3	3.08 ± 0.02	7.48 ± 0.12	5.00
GF3, Green Tea Superior, N/A	2	2.76 ± 0.09	6.10 ± 0.06	5.20
GF4, Green Tea, N/A	2	2.90 ± 0.05	3.95 ± 0.05	2.00
GF5, Green Tea, N/A	2	2.95 ± 0.06	6.13 ± 0.09	3.78
Green loose (GL) tea				
GL1, Kukicha Organic, Japan	2	0.34 ± 0.01	0.78 ± 0.01	6.21
GL2, Sencha Yamato, Japan	2	0.86 ± 0.01	0.93 ± 0.02	5.48
GL3, Yunnan Green, China	3	0.71 ± 0.02	1.48 ± 0.07	4.38
GL4, Earl Green, China	2	1.43 ± 0.05	1.79 ± 0.01	3.65
GL5, China Gunpowder Temple of Heaven, China	2	0.82 ± 0.03	2.43 ± 0.01	3.11
GL6, Jade Dragon Wings Organic, China	2	1.29 ± 0.03	2.23 ± 0.10	3.84
GL7, Korea Mystic Green Organic, South Korea	2	0.96 ± 0.01	3.74 ± 0.19	4.02
GL8, Premium Shincha Gyokuro Okumidori Kirishima 2021, Japan	2	0.67 ± 0.03	0.51 ± 0.01	20.09
GL9, Genmaicha, Japan	2	0.57 ± 0.02	1.31 ± 0.08	3.65

^1^ Teas brand names can be found in Appendix B. ^2^ Number of measurements. ^3^ Values are averages ± standard deviation (SD). ^4^ N/A—not available.

## Data Availability

The data are available upon request to the authors.

## Sample Availability

Samples of the tea are available from the authors.

## Appendix A. Determination of Limit of Detection

Fluoride: The samples were spiked by adding F^–^ concentration of 0.20 µg/mL to bring the concentration in low-level samples within the linear working range of the electrode. Therefore, this F^–^ concentration was also added to the blank samples (*C*_bl-F_) when determining the limit of detection (LOD). Calibration curve technique: The LOD was estimated from the calibration function for a signal equal to the net signal of a difference between the calibration standard and blank samples and three times the standard deviation of this difference [49]. This resulted from at least 10 independent reagent blank solutions, each measured twice on the same day. The LOD determined in this way was 0.02 mg/L, which is consistent with the manufacturer’s stated LOD (0.02 mg/L) [50]. Standard addition technique: The LOD was determined as a net signal of a difference between mean F^–^ concentration determined in two sets of at least ten consecutive measurements of blank plus three times the standard deviation of the difference [47]. The LOD determined in this way was found to be 0.013 mg/L. The LOD is lower than LOD reported by the manufacturer [50], because the mean fluoride concentration in blank samples is not affected by daily electrode drift, as in the calibration curve method.

Aluminium: The LOD was estimated from the calibration function for a signal equal to the net signal of blank and three times its standard deviation (SD_bl_) [49]. This resulted from the analysis of 10 independent reagent blank solutions, each measured once on the same day. The LOD of aluminium was determined to be 0.0013 mg/L.

## Appendix B. List of Teas According to Brand

1001 Cvet (GF5), Ahmad Tea (BF2, BF6, GF2), Sir Winston Tea (BF3, BF9, GF3), Spar (GF4, BL11), Teahouse Cha (BL1, BL2, BL3, BL4, BL5, BL6, BL7, BL8, BL9, BL10, GL1, GL2, GL3, GL4, GL5, GL6, GL7, GL8, GL9), Teekanne (BF4, BF8, BF10, GF1), Twinings (BF1, BF5, BF7).
